# NF90/ILF3 is a transcription factor that promotes proliferation over differentiation by hierarchical regulation in K562 erythroleukemia cells

**DOI:** 10.1371/journal.pone.0193126

**Published:** 2018-03-28

**Authors:** Ting-Hsuan Wu, Lingfang Shi, Jessika Adrian, Minyi Shi, Ramesh V. Nair, Michael P. Snyder, Peter N. Kao

**Affiliations:** 1 Pulmonary and Critical Care Medicine, Stanford University School of Medicine, Stanford, California, United States of America; 2 Biomedical Informatics, Stanford University School of Medicine, Stanford, California, United States of America; 3 Department of Genetics, Stanford University School of Medicine, Stanford, California, United States of America; 4 Stanford Center for Genomics and Personalized Medicine, Stanford University School of Medicine, Palo Alto, California, United States of America; Yeshiva University Albert Einstein College of Medicine, UNITED STATES

## Abstract

NF90 and splice variant NF110 are DNA- and RNA-binding proteins encoded by the Interleukin enhancer-binding factor 3 (*ILF3*) gene that have been established to regulate RNA splicing, stabilization and export. The roles of NF90 and NF110 in regulating transcription as chromatin-interacting proteins have not been comprehensively characterized. Here, chromatin immunoprecipitation followed by deep sequencing (ChIP-seq) identified 9,081 genomic sites specifically occupied by NF90/NF110 in K562 cells. One third of NF90/NF110 peaks occurred at promoters of annotated genes. NF90/NF110 occupancy colocalized with chromatin marks associated with active promoters and strong enhancers. Comparison with 150 ENCODE ChIP-seq experiments revealed that NF90/NF110 clustered with transcription factors exhibiting preference for promoters over enhancers (*POLR2A*, *MYC*, *YY1*). Differential gene expression analysis following shRNA knockdown of NF90/NF110 in K562 cells revealed that NF90/NF110 activates transcription factors that drive growth and proliferation (*EGR1*, *MYC*), while attenuating differentiation along the erythroid lineage (*KLF1*). NF90/NF110 associates with chromatin to hierarchically regulate transcription factors that promote proliferation and suppress differentiation.

## Introduction

Eukaryotic gene expression depends on tight and dynamic regulation of RNA transcription. Transcription of RNA occurs pervasively, and is critically modulated by nucleic acid-binding proteins at the levels of epigenetic control of chromatin landscape, transcription of the genome from DNA into RNA, and regulated stability of the resulting transcript [1]. Dynamic regulation of gene expression confers organismal diversity [2], cell-type complexity [3], and underlies the immediate early response of a cell upon activation by external stimuli [4].

Chromatin regulation is crucial for control of eukaryotic RNA transcription. Chromatin is packed into repeating units of nucleosomes [5]. Binding of transcription factors to DNA is regulated by chromatin remodelers that unwind nucleosomes to expose *cis*-regulatory elements [6, 7]. Open chromatin regions may be transcribed. Significant portions of the genome give rise to RNA. The eukaryotic transcriptome extends beyond annotated protein-coding genes to include non-coding RNAs (ncRNAs) [8, 9]. Functional regulatory elements, such as enhancers, are also transcribed to produce enhancer RNAs (eRNAs) [10]. Analysis of transcription initiation revealed that promoters and enhancers share a unified architecture of tightly spaced divergent transcription start site (TSS) pairs, and bi-directional production of transcripts [11].

Stability of the RNA product is regulated after transcription. At promoters for protein-coding genes, the sense transcript is typically stable, while the upstream antisense RNA (uaRNA) is susceptible to rapid degradation [12]. A pair of stable transcripts may also be produced at bidirectional promoters if each transcript respectively encodes mRNA, or if an mRNA is accompanied by a long non-coding RNA (lncRNA) [13].

DNA- and RNA-binding proteins (DRBPs) interact with diverse classes of nucleic acids and regulate RNA transcription at multiple levels. RNA-binding proteins are often studied as regulators of protein translation [14]. However, recent studies of dual DNA- and RNA-binding proteins demonstrated their multifunctional roles in modulating gene expression [15, 16].

Nuclear Factor 90 (NF90) is a protein encoded by the Interleukin enhancer-binding factor 3 (*ILF3*) gene first cloned as part of a multi-protein complex purified by DNA affinity chromatography from activated Jurkat T cell nuclear extract based on inducible and specific binding to the Nuclear Factor of Activated T cell (NF-AT) target sequence in the IL-2 promoter [17]. The proteins that formed a heterodimer comprising the inducible NF-AT DNA-binding complex were named Nuclear Factor 90 (NF90, *ILF3*) and Nuclear Factor 45 (NF45, *ILF2*) [18].

Subsequent to their initial discovery, NF90 and NF45 have been identified in diverse cellular processes including DNA-break repair [19], cell cycle regulation [20–23], cell growth and proliferation [22, 24–30]. NF90 and its C-terminally extended isoform, NF110, are generated from differentially spliced transcripts of the *ILF3* gene [31, 32]. Both NF90 and NF110 contain a zinc finger domain that mediates hetero-dimerization with NF45 [33], two double-stranded RNA binding domains, and an arginine-glycine-glycine rich RGG domain that interacts with nucleic acids. NF110 contains an additional GQSY region that can interact with nucleic acids [32]. NF90 participates in regulation of mRNA nuclear export and translation [34–37], microRNA processing [38, 39], and as a host factor in viral replication [40–45].

NF90 and NF110 have been extensively studied as a RNA-binding proteins. However, the original purification of NF90 using DNA affinity chromatography and subsequent ChIP experiments indicated a role for NF90/NF110 in gene regulation through its capacity to bind DNA [46–48]. Both NF90 and NF110 were shown to associate with chromatin and to stimulate transcription [49].

Here, we performed ChIP-seq of NF90/NF110 and integrated available ENCODE data in K562 cells to characterize NF90/NF110 chromatin occupancy and regulation of gene expression. We reveal a novel role for NF90 and NF110 as dual DNA- and RNA-binding proteins and important regulators of chromatin, RNA transcription, and transcript stability.

## Results

### NF90/NF110 associates with the genome extensively

To analyze genome-wide occupancy of NF90/NF110, we performed ChIP-seq in K562 chronic myelogenous leukemia cells, a ‘Tier 1’ cell line prioritized by the Encyclopedia of DNA Elements (ENCODE) Project. Abundant data mapped onto the Human Genome version 19 (hg19) reference genome from K562 experiments deposited by various ENCODE-affiliated groups allowed us to integrate our ChIP-seq analysis with studies on chromatin landscape, transcription factor occupancy, and gene expression analysis of the K562 genome to elucidate the role of NF90/NF110 in regulating gene expression in the context of diverse combinatorial events on the chromatin.

**Fig 1:**
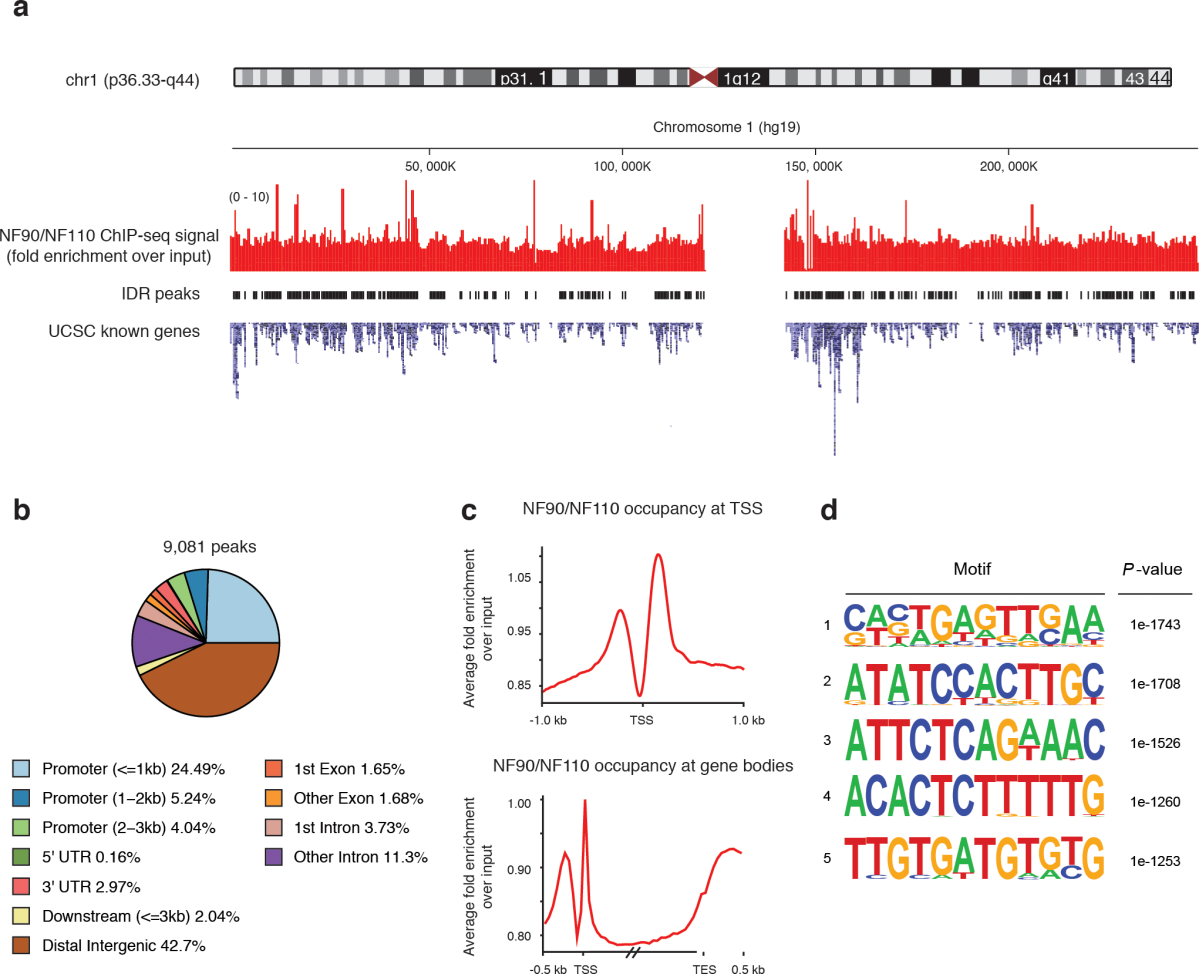
ChIP-seq reveals extensive NF90/NF110 occupancy along the genome. **(a)** ChIP-seq signal (fold enrichment over input) of NF90/NF110 shown along chromosome one, along peaks called using Irreproducibility Discovery Rate (IDR) between biological replicates. UCSC known genes and transcripts are aligned. **(b)** Distribution of annotated features near NF90/NF110 binding sites. **(c)** Average binding profile of NF90/NF110 computed using all annotated transcription start sites (TSS). For gene bodies, all genes were scaled to a 1.5 kb length; the 0.5 kb flanking regions were not scaled. **(d)** Motif analysis (HOMER) based on NF90/NF110 ChIP-seq results in K562 cells showing enriched motifs in NF90/NF110 peaks. Shown: motifs ranked top 5 by statistical significance.

ChIP-seq revealed extensive occupancy of NF90/NF110 on the genome. Over 9,000 recovered NF90/NF110 peaks were consistent across Irreproducible Discovery Rate (IDR) analysis of biological replicates (**Fig 1A**) (**S1 Table**). We observed occupancy near annotated transcription start sites (TSS) (**Fig 1C**), with over one third of peaks occurring at upstream promoter regions (**Fig 1B**). A plurality of peaks occurred in distal intergenic regions (**Fig 1B**). Examining the average NF90/NF110 occupancy profile near all TSS, we found a conserved pattern featuring bimodal occupancy of NF90/NF110 with peaks flanking the TSS, with a stronger peak typically occurring downstream of the TSS. Along the entire transcribed loci of gene bodies, there is enrichment both near the TSS, and the transcription end site (TES) (**Fig 1C**).

### NF90/NF110 peaks are enriched for the NF-AT sequence

We performed *de novo* motif discovery on NF90/NF110 IDR peaks and recovered several motifs that were statistically enriched (**Fig 1D**). Notably, the 5’–CTCTTTTT– 3’ (reverse complement: 5’–AAAAAGAG– 3’) motif was discovered to be highly enriched (*P* = 1e-1260). The reverse complement of this motif bears similarity to the purine-rich Nuclear Factor of Activated T cell (NF-AT)/ antigen receptor response element 2 (ARRE-2) target sequence in the Interleukin 2 (IL-2) promoter (5’–GAGGAAAAACTGTTTCATACAGAAGGCGT– 3’). This is consistent with the original purification of NF90/NF110 from activated T cell nuclei using DNA affinity chromatography to the ARRE-2 target sequence [17, 18].

### NF90/NF110 occupancy is enriched at active promoters and enhancers

The exact occupancy pattern of a transcription factor depends on genomic accessibility controlled by dynamic chromatin landscape. We sought to study interplay between NF90/NF110 peaks and different chromatin regions bearing distinct epigenetic marks. Previously, Ernst and Kellis demonstrated that chromatin landscapes of nine human cell types could be characterized into 15 distinct states by analyzing 14 genome-wide chromatin tracks using a hidden Markov model [50]. Several histone modification marks were used, as well as RNA polymerase II, and *CTCF*, a sequence-specific insulator protein. Chromatin states were annotated into six broad classes, including: promoter, enhancer, insulator, transcribed, repressed, and inactive states.

**Fig 2:**
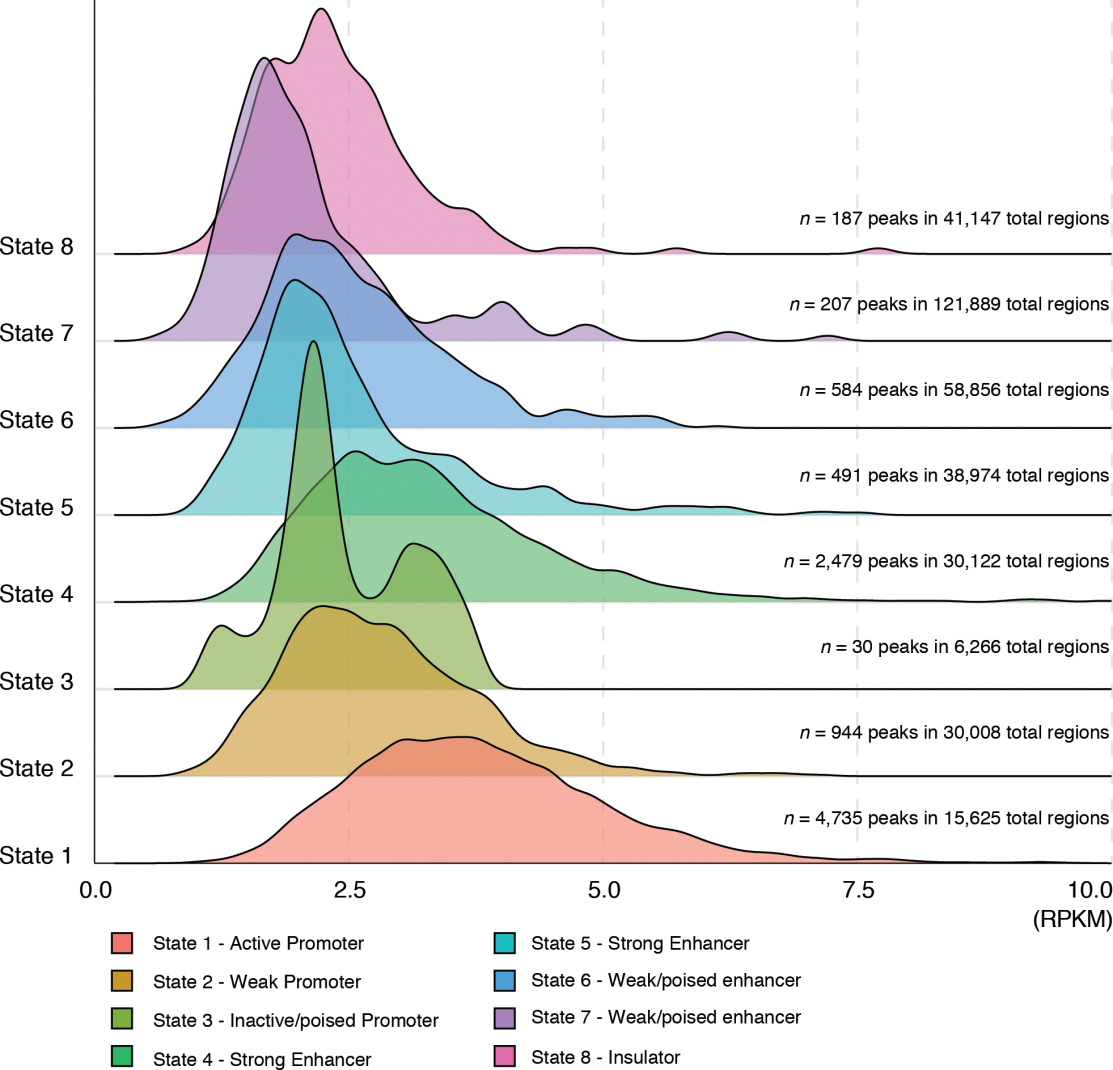
NF90/NF110 relative enrichment in different chromatin states. IDR peaks called from NF90/NF110 ChIP-seq experiment in K562 were sorted according to the chromatin state they resided in. The segmented peaks for each of 15 chromatin states were then used to query the ChIP-seq read files to count the number of reads to obtain affinity information for each peak. The resulting distribution of NF90/NF110 occupancy frequencies in different chromatin states were plotted as a histogram. *x*-axis: Reads Per Kilobase of transcript per Million mapped reads (RPKM). *y*-axis: 8 of 15 chromatin states in which majority of NF90/NF110 peaks resided in.

We analyzed NF90/NF110 occupancy frequencies in each chromatin state. Over 9,000 NF90/NF110 ChIP-seq peaks were sorted based on their underlying chromatin state annotation. Substantial NF90/NF110 occupancy was observed in 8 of the 15 chromatin states (**Fig 2**and **S1 Fig**). The majority of NF90/NF110 peaks were found in state 1 –active promoter (4,735 peaks), followed by state 4 –strong enhancer (2,479 peaks). Of all active promoter (State 1) regions annotated for the K562 genome, 30% were occupied by NF90/NF110 (**Fig 2**). The predominant occupancy of NF90/NF110 at active promoters (State 1) is consistent with our finding that NF90/NF110 peaks frequently occur near annotated TSS (**Fig 1B**).

NF90/NF110 occupied active promoters and strong enhancers with higher frequency compared to other chromatin states. NF90/NF110 peaks in active promoters (State 1) exhibited the highest occupancy frequencies (**Fig 2**, RPKM on *x*-axis), followed by those in strong enhancers (State 4), and weak promoters (State 2). The normal shape of the histogram in these open chromatin regions indicates a continuous range of occupancy frequencies of NF90/NF110, suggesting nonspecific and promiscuous association of the protein at these permissive chromatin states [51]. This is consistent with previous studies that found motif depletion and nonspecific occupancy of transcription factors in permissive chromatin regions [52].

### NF90/NF110 occupancy colocalizes with selective histone marks at active promoters and enhancers

**Fig 3:**
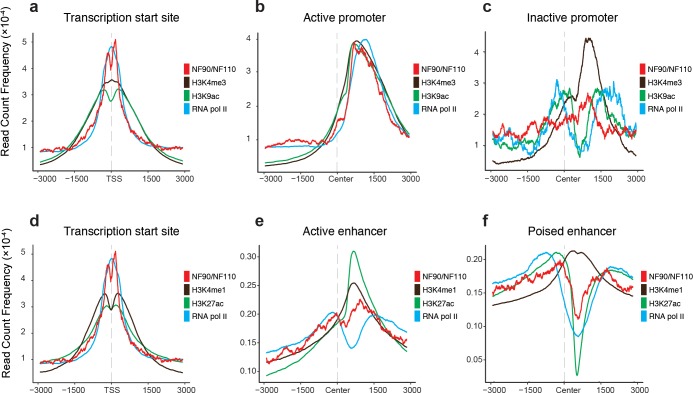
NF90/NF110 colocalization with specific histone marks at active promoters and enhancers. **a-c.** Average occupancy profile of NF90/NF110, H3K4me3, H3K9ac, and RNA pol II at **(a)** transcription start sites, **(b)** active promoters, and **(c)** inactive promoters. **d-f**. Average occupancy profile of NF90/NF110, H3K4me1, H3K27ac, and RNA pol II at **(d)** transcription start sites, **(e)** strong enhancers, and **(f)** poised enhancers. Transcription start sites retrieved from UCSC Known Genes database. Active promoters, inactive promoters, strong enhancers and poised enhancers from Ernst *et al*. *x*-axis: relative position near TSS. *y*-axis: read count frequency of tag within region.

Combinatorial histone marks partitioned the K562 genome by transcriptional activity [50, 52], and may define distinct chromatin states [53]. To investigate if NF90/NF110 physically colocalized with the key histone marks that respectively defined promoter and enhancer states, we accessed histone modification ChIP-seq experiments performed on K562 deposited in ENCODE. The average occupancy profiles of NF90/NF110 alongside key histone marks were graphed at selected chromatin states (**Fig 3**).

We aligned NF90/NF110 ChIP-seq occupancy with the histone marks enriched for active promoter occupancy: RNA pol II, H3K9ac, and H3K4me3 (**Fig 3A–3C**). Histone modifications H3K4me3 and H3K9ac both showed substantial occupancy, as expected; NF90/NF110 occupancy frequency was comparable to RNA pol II at transcription start sites (**Fig 3A**). The shapes of occupancy curves of H3K9ac and NF90/NF110 were similar, as both exhibited bimodal occupancy (**Fig 3A**). At active promoters, the average occupancy profiles of NF90/NF110, H3K4me3, H3K9ac, and RNA pol II showed colocalization with comparable occupancy frequencies. All tracks consistently peaked approximately 1000 bp downstream of the defined centers of active promoter regions (**Fig 3B**). In contrast, when we examined average occupancy profiles at inactive promoters, we found decreased occupancy of NF90/NF110, H3K9ac and RNA pol II (**Fig 3C**). At inactive promoters, only H3K4me3 exhibited a discernible peak (**Fig 3C**).

Next, we aligned NF90/NF110 ChIP-seq occupancy with the histone marks enriched for strong enhancers: RNA pol II, H3K27ac, and H3K4me1 (**Fig 3D–3F**). NF90/NF110 occupancy colocalized with both H3K27ac and H3K4me1, with all three tracks exhibiting bimodal occupancy flanking the TSS (**Fig 3D**). At strong enhancers, NF90/NF110 colocalized with H3K27ac and H3K4me1, with H3K27ac showing strongest occupancy, and NF90/NF110 showing occupancy frequency comparable to H3K4me1 (**Fig 3E**). All tracks consistently peaked approximately 1000 bp downstream of the center of the strong enhancers. In contrast, at poised enhancers, NF90/NF110 occupancy colocalized with H3K27ac and both tracks exhibited a substantial decrease approximately 500 bp downstream of the center of the poised enhancers. H3K4me1 still showed occupancy at poised enhancers with a defined peak (**Fig 3F**), demonstrating more promiscuous occupancy, consistent with literature describing broader association of H3K4me1 with both active and poised enhancers [54].

### NF90/NF110 occupancy pattern by chromatin state clusters with sequence-specific transcription factors

Open chromatin is generally permissive to transcription factor occupancy. However, distinct classes of regulatory factors may show different preferences for different types of open chromatin [52].

To investigate if the chromatin state preference of NF90/NF110 may cluster with similar regulatory factors, we accessed 150 datasets of transcription factor (TF) ChIP-seq peaks performed in K562 cells that were generated by the ENCODE analysis working group using a uniform processing pipeline (**S2 Table**). We computed the relative enrichment of all 150 TF peaks as well as NF90/NF110 ChIP-seq. An unsupervised *k*-means algorithm was used to cluster regulatory factors into four distinct classes.

**Fig 4:**
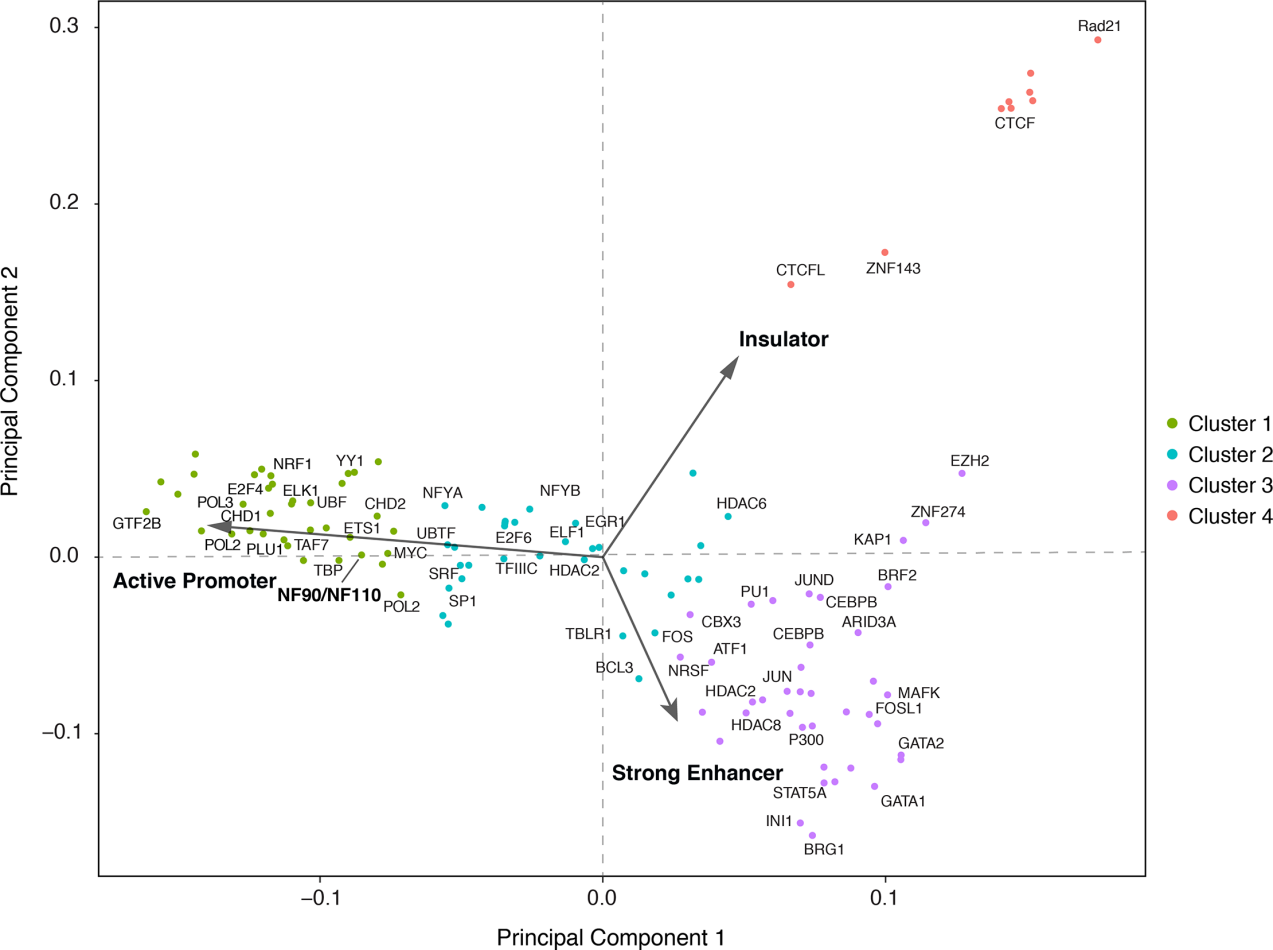
Clustering of transcription factors based on relative enrichment for chromatin states. 150 datasets of transcription factor ChIP-seq peaks based on ENCODE data production centers processed through a uniform processing pipeline were retrieved. 100 unique regulatory factors, including generic and sequence-specific factors were retrieved for the K562 line to supplement the NF90/NF110 ChIP-seq data. For a given transcription factor ChIP-seq peak set, the relative enrichment in different chromatin states was computed. Enrichments were then row-normalized by the largest enrichment values for each experiment. *K*-means clustering with *K* = 4 produced the clusters graphed here using principal component analysis (PCA). The major eigenvectors for the original dataset is depicted in blue arrows.

Principal component analysis (PCA) revealed that the four classes are well separated along the first two principal components, indicating that much of the variance is explained by a relative enrichment among a few of the chromatin states (**Fig 4**). The major eigenvectors plotted show that most variance among regulatory factors in the dataset is explained by the relative enrichment in: State 1 –active promoter, State 4 –strong enhancer, and State 8 –insulator (**Fig 4**). NF90/NF110 occupancy was strongly biased towards active promoters, consistent with our previous findings (**Fig 2**). NF90/NF110 chromatin occupancy most resembled other sequence-specific transcription factors in Cluster 1. Notably, NF90/NF110 clusters closely with the proto-oncogenic transcription factors *MYC* and *ETS1*. In contrast, the regulatory factors in Cluster 3, such as the histone acetyltransferase *EP300*, showed preference for strong enhancers, and may represent general coactivators. NF90/NF110 is well separated from the regulators in Cluster 4, primarily *CTCF* insulator proteins with transcriptional repression activity.

### NF90/NF110 occupancy at divergent transcription initiation regions is enriched for transcript stability

A substantial fraction of the eukaryotic genome is transcribed, with bidirectional initiation resulting in transcripts of varying stability. Core *et al*. previously characterized all pairs of TSS found along the K562 genome by measuring *de novo* transcription using a variation of Global Run-On Sequencing that enriches for 5’-capped RNAs (GRO-cap). The GRO-cap signal is selective for transcription initiation events, while sustained GRO-seq tends to detect stable transcripts. Thus, the stability of transcripts at each TSS pair could be characterized as stable or unstable using a hidden Markov model [11].

**Fig 5:**
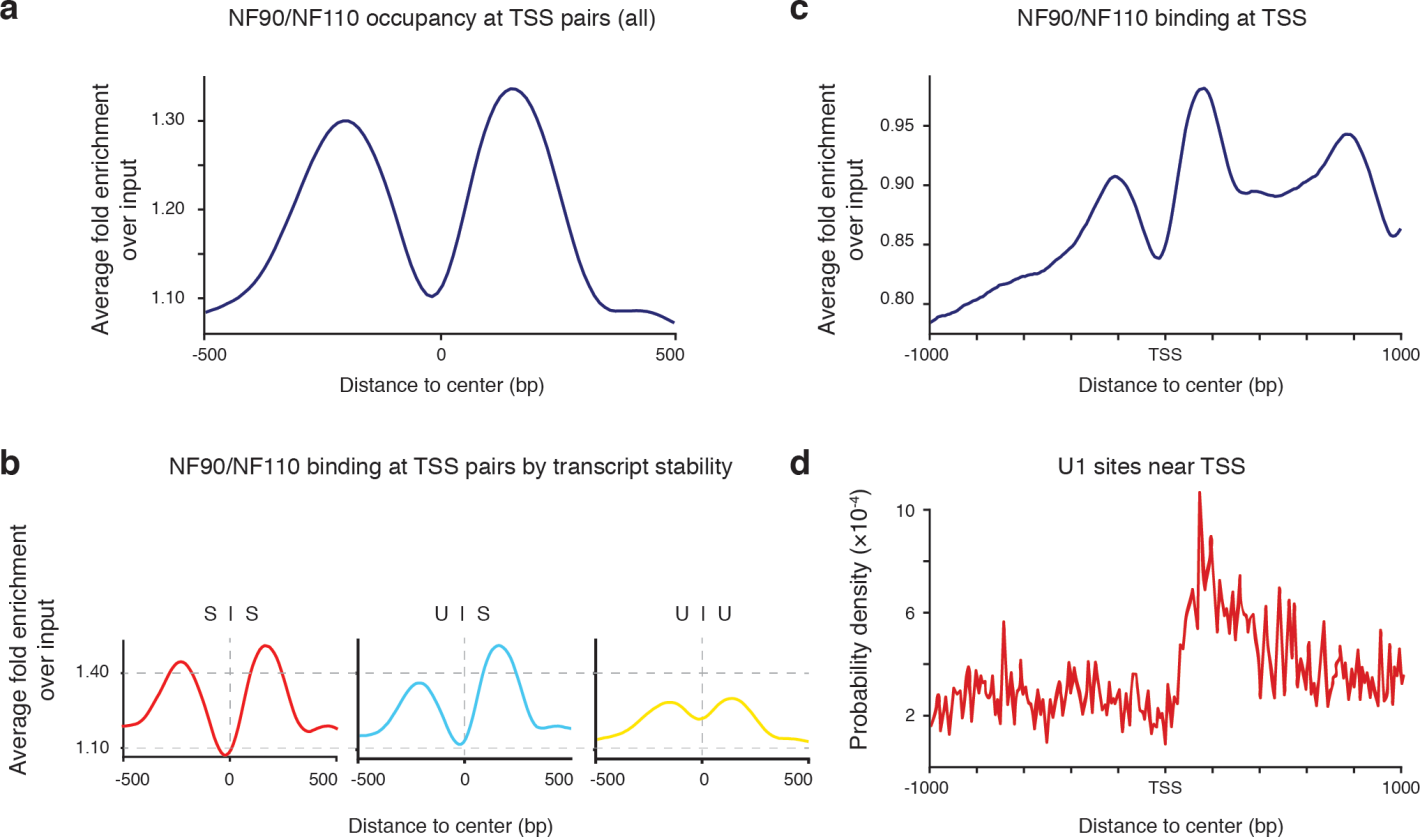
NF90/NF110 occupancy at divergent transcription initiation regions is enriched for transcript stability. **(a)** Average occupancy profile of NF90/NF110 ChIP-seq peaks at all transcription start site (TSS) pairs. **(b)** Average occupancy profile of NF90/NF110 -seq peaks at transcription start site (TSS) pairs sorted by transcript stability parity. SS = stable–stable, US = unstable–stable, UU = unstable–unstable. **(c)** Average occupancy profile of NF90/NF110 ChIP-seq peaks centered at TSS with 1kb flanking regions. **(d)** Local motif enrichment analysis of the U1 SS5 splice sequence centered at TSS with 1kb flanking regions. TSS pair coordinates from Core *et al*.

To investigate if NF90/NF110 occupancy is persistent at TSS pairs that define divergent transcription initiation, we retrieved the coordinates for all *de novo* TSS pairs in the K562 genome discovered using GRO-cap. The average occupancy profile of NF90/NF110 at *de novo* TSS pairs was bimodal with peaks flanking the center of the region (**Fig 5A**), resembling what we found at annotated TSS (**Fig 1C**).

Bimodal occupancy of NF90/NF110 at TSS pairs suggests selective association with individual transcripts rather than shared machinery. To investigate whether NF90/NF110 occupancy frequency at individual transcripts correlated with stability, we sub-setted the original collection of TSS pairs into: stable-stable, unstable-stable, and unstable-unstable transcript pairs, as originally described by Core *et al*. NF90/NF110 occupied stable-stable TSS pairs equally well, replicating the bimodal enrichment found in all TSS pairs (**Fig 5B**). The NF90/NF110 occupancy profile at unstable-stable TSS pairs demonstrated preferential enrichment at stable transcripts, whereas at unstable-unstable TSS pairs there was equal depletion of NF90/NF110 occupancy (**Fig 5B**).

DNA sequence is known to influence the stability of the resulting RNA transcript [55]. The U1 splicing complex directed by the U1 small nuclear ribonucleoprotein (snRNP) to 5’ splice sites (SS5) may protect the stability of the nascent transcript through productive elongation. Previously, NF90/NF110 was shown to interact with multiple components of the spliceosome [56]. We investigated if NF90/NF110 occupancy preference at stable transcripts was related to presence of the U1 SS5 motif. All TSS were searched for enrichment of the U1 SS5 motif, and this enrichment was compared to the relative occupancy of NF90/NF110 near TSS. We observed NF90/NF110 peaks at 200 and 800 bp downstream of TSS (**Fig 5C**). The first NF90/NF110 peak colocalizes with enrichment of the U1 SS5 site approximately 200 bp downstream of the TSS (**Fig 5C and 5D**). These observations suggest that NF90/NF110 regulates transcript stability by chromatin occupancy at the U1 SS5 site and another potential site 800 bp downstream of TSS, through recruitment of splicing machinery to the RNA transcript.

### NF90/NF110 regulates transcription factors that promote K562 proliferation over differentiation

To investigate the functional role of NF90/NF110 in regulating transcription, we accessed ENCODE RNA-seq data in K562 cells treated with shRNA directed against NF90/NF110. We accessed this ENCODE published dataset to measure the global effects on the transcriptome of K562 cells upon NF90/NF110 knockdown, and intersected these differentially expressed genes with our NF90/NF110 ChIP-seq dataset to identify genes under transcriptional regulation by NF90/NF110.

**Fig 6:**
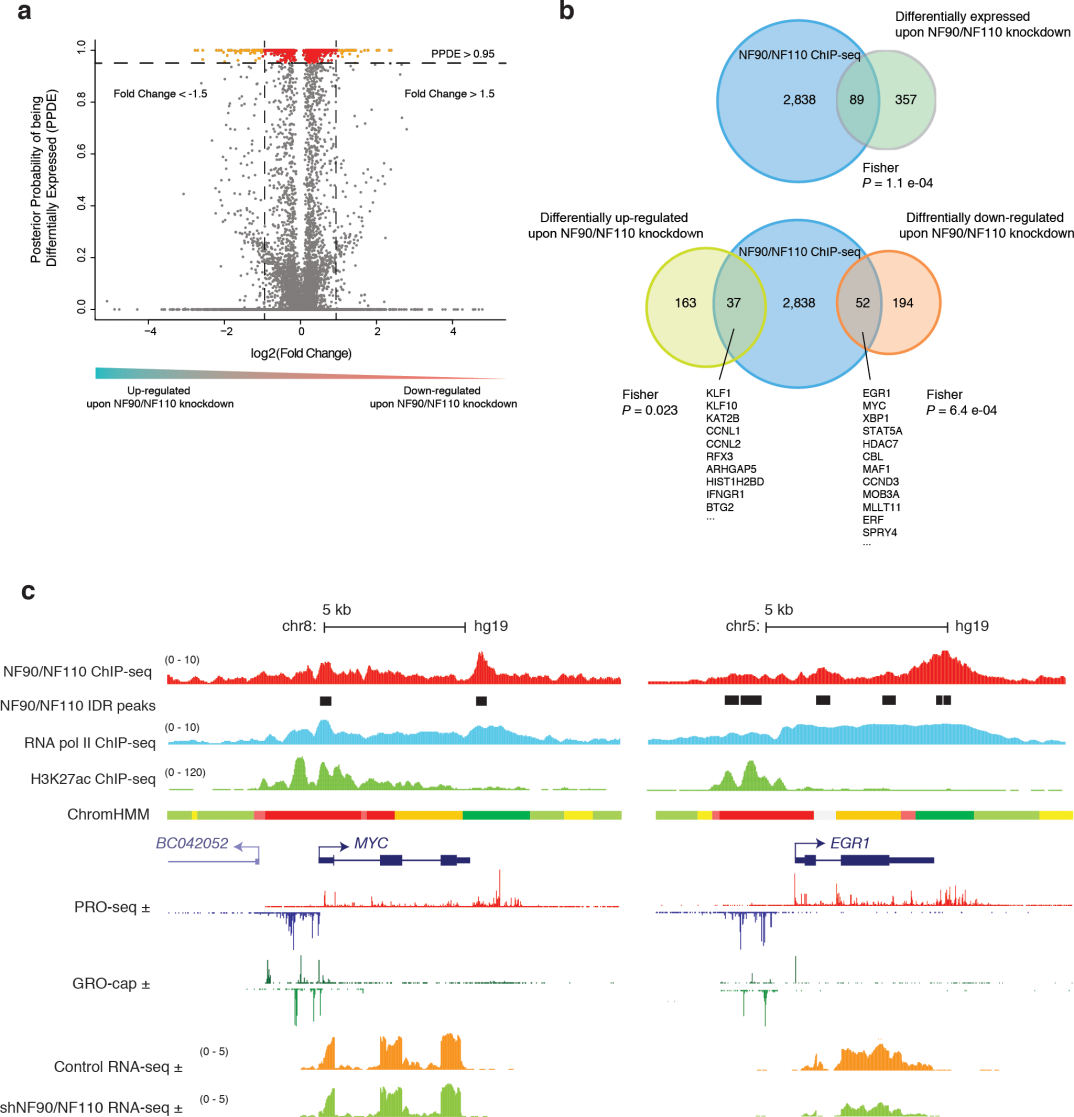
NF90/NF110 regulates transcription factors that promote K562 proliferation over differentiation. **(a)** Volcano plot demonstrating statistically significant changes in gene expression between control and NF90/NF110 knockdown in K562. *x*-axis: log fold change in shRNA against NF90/NF110 compared to control. *y-*axis: posterior probability of being differentially expressed (PPDE). Each dot is a gene; red: PPDE > 0.95; orange: PPDE > 0.95 and fold change > 1.5. **(b)** Venn diagram analyzing overlap between NF90/NF110 ChIP-seq genomic targets and differentially expressed gene upon NF90/NF110 knockdown in K562 (*P* = 1.4 e-08). Venn diagram analyzing overlap between NF90/NF110 ChIP-seq genic targets, up-regulated genes upon NF90/NF110 knockdown, and down-regulated genes upon NF90/NF110 knockdown. **(c)** Representative ChIP-seq track alignment of NF90/NF110, RNA pol II, H3K27ac; Chromatin state annotations from ChromHMM (red: active promoter, light red: weak promoter, orange: active enhancer, yellow: weak enhancer, dark green: transcriptional transition/elongation, light green: weakly transcribed); Precision nuclear run-on sequencing (PRO-Seq, a variation of GRO-Seq) and GRO-cap reads in reads per million (RPM); RNA-seq unique reads track alignment of control shRNA, and shRNA against NF90/NF110.

Knockdown of NF90/NF110 resulted in differential expression of 446 genes (**S3 Table**). The posterior probability of differential expression (PPDE) versus log2-transformed Fold Change (FC) was graphed for each gene (**Fig 6A**). There was no obvious preference in directionality of change in gene expression upon NF90/NF110 knockdown; about 20% more genes were down-regulated (246 genes) compared to up-regulated (200 genes) (**Fig 6A**). A list of 2,927 annotated genes for which NF90/NF110 occupancy was discovered in the proximal promoter regions was retrieved. These 2,927 genes were then compared to the 446 differentially expressed genes upon NF90/NF110 knockdown to identify genes that are transcriptionally regulated by NF90/NF110 (**Fig 6B**). The resulting intersection of the NF90/NF110-bound genes with the differentially expressed genes is statistically significant (Fisher’s exact test, *P* = 1.1 e-04) with an integrated set of 89 genes under regulation by NF90/NF110 (**Fig 6B**, **S4 Table**).

We further interrogated the effect of NF90/NF110 transcriptional regulation by separately intersecting the differentially down-regulated or up-regulated genes upon NF90/NF110 knockdown, with the NF90/NF110 ChIP-seq promoter peaks. Out of 200 genes that were differentially up-regulated upon NF90/NF110 knockdown, NF90/NF110 occupancy was found in the promoters of 37 genes (**Fig 6B**, Fisher’s exact test, *P* = 0.023). These genes are under negative regulation by NF90/NF110. Out of 246 genes that were differentially down-regulated upon NF90/NF110 knockdown, NF90/NF110 occupancy was found in the promoters of 52 genes (**Fig 6B**, Fisher’s exact test, *P* = 6.4 e-04). These genes are under positive regulation by NF90/NF110.

Gene set enrichment analysis (GSEA) revealed the functional profile of genes under regulation by NF90/NF110. The set of 37 genes under negative regulation by NF90/NF110 was significantly enriched for Hallmark of Heme Metabolism (**Table 1**, **S4 Table**). Krueppel-like factor 1 (*KLF1*) is a transcription factor that specifies hematopoietic differentiation and is required for erythroid maturation. The promoter of *KLF1* is bound by NF90/NF110, and *KLF1* RNA is significantly up-regulated upon NF90/NF110 knockdown (PPDE = 1, FC = 0.67) (**S4 Table**). GSEA of genes under positive regulation by NF90/NF110 revealed statistically significant overrepresentation of proto-oncogenes involved in Positive Regulation of Epithelial Cell Proliferation (**Table 2**, **S4 Table**). Moreover, NF90/NF110 regulation of genes involved in TNFα Signaling via NF-κB and Cellular Response to Stress (**Table 2, S4 Table**) indicate that NF90/NF110 is involved in the rapid transcriptional response following extracellular stimulation, consistent with its described role in regulating inducible expression of IL-2 upon T-cell stimulation [21, 46], as well as regulation of *FOS* immediate early expression upon stimulation [48].

**Table 1:** Gene set enrichment analysis (GSEA) of genes under negative regulation by NF90/NF110.

Gene Set Name	Genes in Gene Set (*K*)	Genes in Overlap (*k*)	*k/K*	FDR q-value
Hallmark of Heme Metabolism	200	16	0.08	1.20E-26

FDR, False Discovery Rate.

**Table 2:** Gene set enrichment analysis (GSEA) of genes under positive regulation by NF90/NF110.

Gene Set Name	Genes in Gene Set (*K*)	Genes in Overlap (*k*)	*k/K*	FDR q-value
Positive Regulation of Epithelial Cell Proliferation (GO)	154	7	0.0455	2.33E-07
Hallmark of TNFα Signaling via NF-κB	200	7	0.035	7.32E-07
Cellular Response to Stress (GO)	1565	12	0.0077	3.90E-06
Regulation of Epithelial Cell Proliferation (GO)	285	7	0.0246	4.29E-06
Regulation of Cell Death (GO)	1472	10	0.0068	1.72E-04

GO, Gene Ontology; FDR, False Discovery Rate.

Specific examples of NF90/NF110 chromatin occupancy at the *MYC* and *EGR1* loci are shown in **Fig 6C**. At both promoters, divergent transcription is evidenced by equivalent GRO-cap signals on both the plus and minus strands. In contrast, the PRO-seq signals extended farther on the plus strands that encode the genes, compared to the upstream antisense minus strands. NF90/NF110 chromatin occupancy was greater on the plus strands at TSS and was sustained through the gene bodies, than on the minus strands. NF90/NF110 chromatin occupancy throughout the gene body paralleled RNA Pol II chromatin occupancy. Distinct from RNA Pol II, NF90/NF110 chromatin occupancy was enriched at the Transcription End Sites (TES) (**Figs 6C** and **1C**). *MYC* and *EGR1* are transcriptional targets of NF90/NF110, and shRNA knockdown of NF90/NF110 attenuated expression of the mRNA transcripts (**Fig 6C**).

Thus, inspecting the genes under transcriptional regulation by NF90/NF110 revealed that NF90/NF110 activates genes that drive growth and proliferation (*EGR1*, *MYC*), while attenuating differentiation along the erythroid lineage (*KLF1*). NF90/NF110 interacts with chromatin at specific sites to hierarchically regulate transcription factors that promote cell proliferation and suppress differentiation.

## Discussion

### NF90/NF110 as a hierarchical regulator of pluripotency and differentiation

Previous studies of gene regulation by NF90/NF110 as an RNA-binding protein focused mainly on posttranscriptional processes. Here, our ChIP-seq analysis reveals important roles for NF90/NF110 in the transcriptional regulation of transcription factors, pointing to a hierarchical role of NF90/NF110 in the regulatory network of gene expression.

Previously, Ye and colleagues [57] screened RNA-binding proteins necessary for maintenance of pluripotency in embryonic stem cells (ESCs). Targeted disruption of NF90/NF110 and NF45 impaired ESC proliferation and promoted differentiation down embryonic lineages to an epiblast-like state.

Our integrated analysis of genes under regulation by NF90/NF110 supports its role in maintaining pluripotency and suppressing differentiation. The over-representation of proto-oncogenes involved in epithelial cell proliferation under positive regulation by NF90/NF110 suggests a role for NF90/NF110 in regulating growth and proliferation. This is consistent with previous studies of NF90/NF110 involvement in oncogenesis by regulating cell growth, cell cycle, and proliferation [23, 29, 30]. Conversely, genes under negative regulation by NF90/NF110 included Krueppel-like factor 1 (*KLF1*), a critical regulator of hematopoietic development and specifier of the mature phenotype of the erythroid lineage [58].

These discoveries indicate that NF90/NF110 may function both as a positive and negative regulator of gene expression, depending on functional context of the specific promoters occupied by NF90/NF110. The determination of how NF90/NF110 chromatin occupancy affects transcription at specific promoters may be influenced at the levels of combinatorial effects with other transcription factors, or through diverse posttranslational modifications on NF90/NF110 under different cellular contexts. NF90/NF110 is widely phosphorylated *in vivo* (https://www.phosphosite.org/proteinAction.action?id=2947&showAllSites=true), and combinatorial posttranslational modifications on transcription factors have been proposed to specify regulatory effect on their genomic targets [59].

These results suggest a role for NF90/NF110 and its heterodimeric partner NF45 as hierarchical regulators of the push and pull of pluripotency and differentiation by transcriptional control of key transcription factors [57].

### Modulation of NF90/NF110 chromatin occupancy and transcriptional activity

DNA- and RNA-binding proteins (DRBPs) assume an integral role in control of gene expression through versatile interactions with diverse nucleic acids. Consistent with the original biochemical purification of NF90 using DNA-affinity chromatography [17] and subsequent electrophoretic mobility shift assays (EMSA) that showed a sequence-specific DNA binding activity *in vitro* [18], our ChIP-seq results demonstrate that NF90/NF110 associates widely with chromatin *in vivo*.

These results taken together with the body of work on NF90 and NF110 as RNA-binding proteins support the roles of NF90 and NF110 as dual DNA- and RNA-binding proteins. NF90 and NF110 contain two dsRNA binding domains (dsRBDs), and two additional domains that can interact with nucleic acids, both located at the C-terminus: an arginine- and glycine-rich RGG motif, and a GQSY domain [60, 61]. Still lacking is identification of a definitive DNA-binding domain that mediates the wide association between NF90/NF110 and the chromatin described here, and how the DNA- and RNA-binding affinities of NF90/NF110 are regulated.

Functional enhancer elements have recently been recognized to transcribe noncoding RNAs, termed enhancer RNAs [62]. In the CRISPR/Cas9 system, noncoding RNAs complementary to DNA target sequences have been shown to form nuclear ribonucleoprotein particles that localize enzymatic activity to specific loci in the genome [63]. Single stranded RNA may also invade double-stranded DNA forming RNA:DNA hybrid R-loops. Mapping of RNA:DNA hybrids across the human genome revealed extensive occupancy and colocalization with the H3K27ac/H3K4me1 DNAse hypersensitivity epigenetic signature of actively transcribed promoters [64]. Furthermore, there was motif enrichment for GGAA sequences in the RNA components of the RNA:DNA hybrids. Mass spectrometry identified NF45/ILF2 and NF90/ILF3 proteins to be specifically associated with the RNA:DNA hybrids. We propose that nuclear noncoding RNAs may bind to NF90/NF110 and direct its transcriptional activation activity to diverse sites in the genome through RNA:DNA hybridization. The transcription factor YY1 is recognized to bind DNA and RNA and was successfully recruited through tethered RNA to different enhancers in embryonic stem cells [65].

### NF90/NF110 coordination of transcription and translation

NF90/NF110 may coordinate transcription and translation of target genes [66]. Coordinated regulation of transcription and translation by NF90/NF110 on the same target gene has been reported before [21, 34, 35]. NF90/NF110 participates in regulating inducible expression of IL-2 upon T-cell activation on multiple levels. Upon T-cell stimulation, NF90/NF110 associates at the IL-2 proximal promoter dynamically [46], binds to the 3’UTR of the transcribed IL-2 mRNA [35], and mediates nuclear export of mature IL-2 transcript to the cytoplasm through the interaction of NF90/NF110 nuclear export signal with exportin [34]. Diverse mRNAs bound by NF90/NF110 were identified by RNA immunoprecipitation, and NF90/NF110 interaction with these mRNAs modulated their translation [67, 68]. NF90/NF110 regulates hypoxia-inducible expression of vascular endothelial growth factor (VEGF) through binding to the VEGF 3' untranslated region on the transcript [36]. Examining our ChIP-seq data, NF90/NF110 binds to the VEGFA promoter (**S1 Table**).

At the *FOS* locus, NF90/NF110 and NF45 were previously found to associate dynamically along the gene body, correlating spatially and temporally with that of RNA pol II occupancy [48]. Consistent with this study, our ChIP-seq results have shown that in addition to occupancy at active promoters and enhancers, NF90/NF110 is associated extensively along the gene bodies of *EGR1* and *MYC*, which were also identified to be targets of transcriptional activation by NF90/NF110. We note that the transcriptional regulation and extensive intragenic association of NF90/NF110 suggests that NF90/NF110 binds to and regulates post-transcriptional processing of the mRNA synthesized from the specific genomic targets under transcriptional control by NF90/NF110.

Van Nostrand *et al*. previously developed enhanced UV crosslinking and immunoprecipitation (eCLIP) for transcriptome-wide discovery of RNA-binding protein binding targets [69]. The transcriptome-wide interactions of NF90/NF110 were characterized by eCLIP-seq and deposited on ENCODE. NF90/NF110 associated with the transcriptome extensively, binding to over 10,000 transcripts. In nearly all genes where NF90/NF110 occupancy was detected on the genome by ChIP-seq, there was corresponding eCLIP signal indicating NF90/NF110 binding to the resulting transcript. This is consistent with previous studies that have found promiscuous association of NF90/NF110 with its RNA binding partners with no obvious sequence specificity [70].

Our study lends further support to models previously proposed in which NF90/NF110 may regulate transcription and translation of target genes at multiple levels [32, 48]. The extensive chromatin association of NF90/NF110 together with increased occupancy frequency at active promoters suggests a regulatory role in transcription of target genes into RNA. Nascent RNAs may be bound by NF90/NF110 as components of the splicing machinery [71]. NF90/NF110 interactions with mRNAs contribute to stabilization, nuclear export and regulation of translation [67, 68]. Increased NF90/NF110 chromatin occupancy observed at the TES may arise from increased local concentration of NF90/NF110 available to interact with DNA following transcriptional termination and export of nascent RNA from the nucleus. In this model, NF90/NF110 functions analogously to a conveyor belt from chromatin to gene, through RNA transcript to ribosome in dynamic regulation of gene expression.

## Materials and methods

### Data accessed

The following datasets were downloaded from the ENCODE Data Coordination Center (DCC): H3K4me1 ChIP-seq on human K562 (ENCFF730VTO), H3K4me3 on human K562 (ENCFF737AMS), H3K9ac ChIP-seq on human K562 (ENCFF173ULG), H3K27ac ChIP-seq on human K562 (ENCFF044JNJ), POLR2A ChIP-seq on human K562 (ENCFF248IWJ), RNA-seq on K562 cells treated with an shRNA knockdown against ILF3 (ENCFF845BGZ, ENCFF153BJQ), Control shRNA against no target in K562 cells followed by RNA-seq (ENCFF439FIP, ENCFF702YIW). List of 150 ENCODE ChIP-Seq experiments in K562 cells accessed from UCSC Table Browser (http://genome.ucsc.edu/cgi-bin/hgTrackUi?db=hg19&g=wgEncodeAwgTfbsUniform).

### Chromatin immunoprecipitation sequencing (ChIP-seq) analysis

#### Data acquisition and sequencing

All analysis was performed on the Human genome build 19 (GRCh37/hg19) reference genome. ChIP-seq of NF90/NF110 in K562 was performed by the Snyder Data Production Center (DPC) as part of the ENCODE consortium. Antibody used was against NF90/NF110 (mAb DRBP76; BD), and specificity was confirmed by immunoprecipitation followed by SDS-PAGE (**S2 Fig**), then mass spectrometry (**S2 Table**). The antibody exhibited cross-reactivity to NF90 and splice variant NF110. ChIP-seq experiment in K562 protocol, quality control, and preprocessing followed ENCODE standards as part of the ENCODE uniform processing pipeline. [72, 73].

#### Retrieval of genomic coordinates

Genomic coordinates of active promoters, inactive promoters, active enhancers, and poised enhancers in the human genome assembly 19 (hg19) were retrieved from the UCSC Table Browser (http://rohsdb.cmb.usc.edu/GBshape/cgi-bin/hgTables), based on Chromatin state discovery and characterization (ChromHMM) annotation from ENCODE/Broad Institute using ENCODE ChIP-seq data for nine histone modifications in K562 cells performed by Ernst and Kellis, and was accessed from (http://genome.ucsc.edu/cgi-bin/hgFileUi?db=hg19&g=wgEncodeBroadHmm)) (Cell Line = K562; UCSC Accession = wgEncodeEH000790; tableName = wgEncodeBroadHmmK562HMM; md5sum = eb23b5e0970e8d7367bfd784079c088a) [52].

Genomic coordinates of transcription start site (TSS) pairs, including the stability classification for stable-stable, stable-unstable, or unstable-unstable transcripts, were retrieved from the UCSC Table Browser based on PRO-seq and GRO-cap analysis in K562 performed by Core *et al* (http://genome.ucsc.edu/cgi-bin/hgTracks?db=hg19&hubUrl=http://compgen.cshl.edu/GROcap/hub.txt) [11].

#### Data analysis

Genome-wide peak coverage analysis and average occupancy profile for NF90/NF110 ChIP-seq data was performed in R with the ChIPseeker package and DeepTools [74, 75]. Motif enrichment analysis and gene annotation of NF90/NF110 ChIP-seq peaks were computed using Hypergeometric Optimization of Motif EnRichment (HOMER) [76]. NF90/NF110 occupancy frequency in each chromatin state was computed in R with the DiffBind package [77].

#### Relative enrichment and cluster analysis

Relative enrichment of each regulatory factor in different chromatin states were computed essentially as described by Ernst and Kellis [52]. Briefly, to compute the enrichment for a peak call-data set in a specific chromatin state, *s*, we computed the enrichment for transcription factor occupancy as (*a*_*s*_*/b*)/(*c*_*s*_/*d*), where *a*_*s*_ is the total number of bases in a peak call in *s*; *b* is the total number of bases in a peak call; *c*_*s*_ is the total number of bases in *s*; and *d* is the total number of bases for which the segmentation was defined.

For cluster analysis, a single vector of *n* = 15 relative enrichment values for each chromatin state was obtained for *m* = 150 regulatory factors, and concatenated to form a *n*×*m* matrix.

Unsupervised learning was performed in R using the *K*-means function using a range of *K* values. The principal component analysis (PCA) of the clusters were plotted, and *K* = 4 was chosen based on PCA analysis.

### RNA-seq analysis

#### Differential gene expression analysis (EBSeq)

Gene quantification data was accessed from ENCODE: RNA-seq on K562 cells treated with an shRNA knockdown against ILF3 (ENCFF845BGZ, ENCFF153BJQ), Control shRNA against no target in K562 cells followed by RNA-seq (ENCFF439FIP, ENCFF702YIW). Differential gene expression analysis was performed in R using EBSeq package [78], an empirical Bayes hierarchical model for inference in RNA-seq experiments, using false discovery rate of 0.05 to retrieve list of 446 differentially expressed genes upon NF90/NF110 knockdown.

#### Gene set enrichment analysis

Differentially expressed genes were ranked by posterior fold change and enriched for gene sets using The Molecular Signatures Database (MSigDB) and Gene Set Enrichment Analysis (GSEA) tools developed by the Broad Institute.

## Supporting information

S1 Table List of annotated peaks from NF90/NF110 ChIP-seq experiment. NF90/NF110 ChIP-seq peaks in K562 cells from IDR analysis, annotated by determining peak distance from nearest TSS. Peak lists are sorted by category of genomic annotation.
(XLSX)

S2 Table List of 150 ENCODE regulatory factor ChIP-Seq datasets used in cluster analysis. List of 150 datasets of transcription factor ChIP-seq experiments performed in K562 cells based on data from all five ENCODE regulatory factor ChIP-seq production groups from the project inception in 2007 through the ENCODE March 2012 data freeze. The track covers 100 unique regulatory factors, including generic and sequence-specific factors.
(XLSX)

S3 Table Differential gene expression analysis upon NF90/NF110 knockdown in K562 cells. List of 446 differentially expressed genes upon NF90/NF110 knockdown computed by EBSeq. Posterior Probability of being Differentially Expressed (PPDE) is computed, as well as computed Fold Change (FC) of gene expression in control over treatment with shRNA against NF90/NF110.
(XLSX)

S4 Table Genes under regulation by NF90/NF110. List of 89 genes under transcriptional regulation by NF90/NF110 obtained by intersecting NF90/NF110 ChIP-seq genic peaks with genes that were differentially expressed upon NF90/NF110 knockdown. Includes Gene set enrichment analysis (GSEA) analysis as well as gene set matrix indicating membership of each gene in the annotated gene sets.
(XLSX)

S5 Table Immunoprecipitation mass spectrometry (IP-MS) validation of NF90/NF110 antibody used in ChIP-seq experiments. IP followed by mass spectrometry. Briefly, protein was immunoprecipitated from K562 nuclear cell lysates using antibody against NF90/NF110 (mAb DRBP76; BD 612155), and the IP fraction was loaded on a 10% polyacrylamide gel (NuPAGEBis-Tris Gel) and separated with an Invitrogen NuPAGE electrophoresis system. The gel was stained by ColloidialCoomassie G-250 stain, gel fragments corresponding to the bands indicated were excised. Then proteins were trypsinized using in-gel digestion. Digested proteins were analyzed on an Orbitrap Elite mass spectrometer (Thermo Scientific) by the nanoLC-ESI-MS/MS technique. Peptides were identified by the SEQUEST algorithm and filtered with a high confidence threshold (Peptide false discovery rate < 1%, 2 unique peptides per protein minimum, mass error < 10 ppm).
(PDF)

S1 Fig NF90/NF110 relative enrichment in all 15 chromatin states. IDR peaks called from NF90/NF110 ChIP-seq experiment in K562 were sorted according to the chromatin state they resided in. The segmented peaks for each of 15 chromatin states were then used to query the ChIP-seq read files to count the number of reads to obtain affinity information for each peak. The resulting distribution of NF90/NF110 occupancy frequencies in different chromatin states were plotted as a histogram. *x*-axis: Reads Per Kilobase of transcript per Million mapped reads (RPKM). *y*-axis: 15 chromatin states in which NF90/NF110 peaks resided in.
(TIF)

S2 Fig Immunoprecipitation validation of NF90/NF110 antibody used in ChIP-seq experiments. Immunoprecipitation was performed on nuclear extracts from the cell line K562 using antibody against NF90/NF110 (mAb DRBP76; BD 612155). Lane 1: input nuclear lysate. Lane 2: material immunoprecipitated with antibody. Lane 3: material immunoprecipitated using control IgG. Marked bands were excised from gel and subjected to analysis by mass spectrometry. Target molecular weight: 95.338.
(TIF)
