# Supplementary material for: NF90/ILF3 is a transcription factor that promotes proliferation over differentiation by hierarchical regulation in K562 erythroleukemia cells
Source: PLoS One. 2018 Mar 28;13(3):e0193126. doi: 10.1371/journal.pone.0193126 (PMC5873942; doi:10.1371/journal.pone.0193126)
Supplement: S5 Table — IP followed by mass spectrometry. Briefly, protein was immunoprecipitated from K562 nuclear cell lysates using antibody against NF90/NF110 (mAb DRBP76; BD 612155), and the IP fraction was loaded on a 10% polyacrylamide gel (NuPAGEBis-Tris Gel) and separated with an Invitrogen NuPAGE electrophoresis system. The gel was stained by ColloidialCoomassie G-250 stain, gel fragments corresponding to the bands indicated were excised. Then proteins were trypsinized using in-gel digestion. Digested proteins were analyzed on an Orbitrap Elite mass spectrometer (Thermo Scientific) by the nanoLC-ESI-MS/MS technique. Peptides were identified by the SEQUEST algorithm and filtered with a high confidence threshold (Peptide false discovery rate < 1%, 2 unique peptides per protein minimum, mass error < 10 ppm). (PDF) [file pone.0193126.s005.pdf]

Spectrum	Name of Protein	Unique Peptide Counts	Fold Enrichment
ILF3_612155_Band A	Isoform 5 of Interleukin enhancer-binding factor 3 OS=Homo sapiens GN=ILF3 - [ILF3_HUMAN]	2	Not in Control IP
ILF3_612155_Band A	Desmoglein-1 OS=Homo sapiens GN=DSG1 PE=1 SV=2 - [DSG1_HUMAN]	2	Not in Control IP
ILF3_612155_Band A	Isoform 4 of Nucleolar protein 6 OS=Homo sapiens GN=NOL6 - [NOL6_HUMAN]	2	Not in Control IP
ILF3_612155_Band A	Protein RRP5 homolog OS=Homo sapiens GN=PDCD11 PE=1 SV=3 - [RRP5_HUMAN]	2	Not in Control IP
ILF3_612155_Band A	Heat shock cognate 71 kDa protein (Fragment) OS=Homo sapiens GN=HSPA8 PE=1 SV=1 - [E9PN89_HUMAN]	1	Not in Control IP
ILF3_612155_Band A	Isoform 2 of Histone H2A.V OS=Homo sapiens GN=H2AFV - [H2AV_HUMAN]	1	Not in Control IP
ILF3_612155_Band A	Isoform 2 of Acidic leucine-rich nuclear phosphoprotein 32 family member B OS=Homo sapiens GN=ANP32B - [AN32B_HUMAN]	1	Not in Control IP
ILF3_612155_Band A	Isoform 2 of Clathrin heavy chain 1 OS=Homo sapiens GN=CLTC - [CLH1_HUMAN]	1	Not in Control IP
ILF3_612155_Band A	Isoform 5 of Double-stranded RNA-specific adenosine deaminase OS=Homo sapiens GN=ADAR - [DSRAD_HUMAN]	1	Not in Control IP
ILF3_612155_Band A	Filaggrin-2 OS=Homo sapiens GN=FLG2 PE=1 SV=1 - [FILA2_HUMAN]	1	Not in Control IP
ILF3_612155_Band A	Histone H2A type 2-C OS=Homo sapiens GN=HIST2H2AC PE=1 SV=4 - [H2A2C_HUMAN]	1	Not in Control IP
ILF3_612155_Band A	Prolactin-inducible protein OS=Homo sapiens GN=PIP PE=1 SV=1 - [PIP_HUMAN]	1	Not in Control IP
ILF3_612155_Band A	Endoplasmin OS=Homo sapiens GN=HSP90B1 PE=1 SV=1 - [Q96GW1_HUMAN]	1	Not in Control IP
ILF3_612155_Band A	N-acetyltransferase 10 OS=Homo sapiens GN=NAT10 PE=1 SV=1 - [A0A087WV29_HUMAN]	1	Not in Control IP
ILF3_612155_Band A	Cadherin-16 OS=Homo sapiens GN=CDH16 PE=1 SV=1 - [A0A0B4J288_HUMAN]	1	Not in Control IP
ILF3_612155_Band A	U3 small nucleolar RNA-associated protein 14 homolog A (Fragment) OS=Homo sapiens GN=UTP14A PE=1 SV=1 - [X6RJY0_HUMAN]	1	Not in Control IP
ILF3_612155_Band A	Lysozyme OS=Homo sapiens GN=LYZ PE=1 SV=1 - [F8VV32_HUMAN]	1	Not in Control IP
ILF3_612155_Band A	Histone H2A (Fragment) OS=Homo sapiens GN=H2AFJ PE=1 SV=1 - [H0YFX9_HUMAN]	1	Not in Control IP
ILF3_612155_Band A	Trypsin-1 (Fragment) OS=Homo sapiens GN=PRSS1 PE=1 SV=1 - [H0Y8D1_HUMAN]	1	Not in Control IP
ILF3_612155_Band A	Trafficking protein particle complex subunit 9 (Fragment) OS=Homo sapiens GN=TRAPPC9 PE=1 SV=1 - [H0YBR0_HUMAN]	1	Not in Control IP
ILF3_612155_Band A	Protein diaphanous homolog 1 OS=Homo sapiens GN=DIAPH1 PE=1 SV=2 - [H9KV28_HUMAN]	6	6.00
ILF3_612155_Band A	ATP-dependent RNA helicase A OS=Homo sapiens GN=DHX9 PE=1 SV=4 - [DHX9_HUMAN]	9	0.60
ILF3_612155_Band A	Matrin-3 OS=Homo sapiens GN=MATR3 PE=1 SV=1 - [D6REM6_HUMAN]	4	0.50
ILF3_612155_Band A	Isoform Short of Heterogeneous nuclear ribonucleoprotein U OS=Homo sapiens GN=HNRNPU - [HNRPU_HUMAN]	21	0.29
ILF3_612155_Band B	Histone H4 OS=Homo sapiens GN=HIST1H4A PE=1 SV=2 - [H4_HUMAN]	4	Not in Control IP
ILF3_612155_Band B	Complement C3 OS=Homo sapiens GN=C3 PE=1 SV=2 - [CO3_HUMAN]	3	Not in Control IP
ILF3_612155_Band B	Serum albumin OS=Homo sapiens GN=ALB PE=1 SV=1 - [A0A087WWT3_HUMAN]	3	Not in Control IP
ILF3_612155_Band B	Isoform 2 of Histone H2A.V OS=Homo sapiens GN=H2AFV - [H2AV_HUMAN]	2	Not in Control IP
ILF3_612155_Band B	Hemopexin OS=Homo sapiens GN=HPX PE=1 SV=2 - [HEMO_HUMAN]	2	Not in Control IP
ILF3_612155_Band B	Actin, cytoplasmic 1 (Fragment) OS=Homo sapiens GN=ACTB PE=1 SV=5 - [E7EVS6_HUMAN]	2	Not in Control IP
ILF3_612155_Band B	Isoform 5 of Interleukin enhancer-binding factor 3 OS=Homo sapiens GN=ILF3 - [ILF3_HUMAN]	1	Not in Control IP
ILF3_612155_Band B	Isoform 2 of Acidic leucine-rich nuclear phosphoprotein 32 family member B OS=Homo sapiens GN=ANP32B - [AN32B_HUMAN]	1	Not in Control IP
ILF3_612155_Band B	Endoplasmin OS=Homo sapiens GN=HSP90B1 PE=1 SV=1 - [Q96GW1_HUMAN]	1	Not in Control IP
ILF3_612155_Band B	Antithrombin-III OS=Homo sapiens GN=SERPINC1 PE=1 SV=1 - [ANT3_HUMAN]	1	Not in Control IP
ILF3_612155_Band B	Apolipoprotein A-IV OS=Homo sapiens GN=APOA4 PE=1 SV=3 - [APOA4_HUMAN]	1	Not in Control IP
ILF3_612155_Band B	Isoform 2 of DNA polymerase theta OS=Homo sapiens GN=POLQ - [DPOLQ_HUMAN]	1	Not in Control IP
ILF3_612155_Band B	Histone H1t OS=Homo sapiens GN=HIST1H1T PE=2 SV=4 - [H1T_HUMAN]	1	Not in Control IP
ILF3_612155_Band B	Hypermethylated in cancer 2 protein OS=Homo sapiens GN=HIC2 PE=1 SV=2 - [HIC2_HUMAN]	1	Not in Control IP
ILF3_612155_Band B	High mobility group protein HMG-I/HMG-Y OS=Homo sapiens GN=HMGA1 PE=1 SV=3 - [HMGA1_HUMAN]	1	Not in Control IP
ILF3_612155_Band B	Alpha-1-antichymotrypsin OS=Homo sapiens GN=SERPINA3 PE=1 SV=1 - [G3V3A0_HUMAN]	1	Not in Control IP
ILF3_612155_Band B	Elongation factor 1-alpha 1 OS=Homo sapiens GN=EEF1A1 PE=1 SV=1 - [A0A087WVQ9_HUMAN]	1	Not in Control IP
ILF3_612155_Band B	Complement factor B (Fragment) OS=Homo sapiens GN=CFB PE=1 SV=1 - [H7C5H1_HUMAN]	1	Not in Control IP
ILF3_612155_Band B	Protein AMBP (Fragment) OS=Homo sapiens GN=AMBP PE=1 SV=1 - [S4R471_HUMAN]	1	Not in Control IP
ILF3_612155_Band B	Complement factor H OS=Homo sapiens GN=CFH PE=1 SV=1 - [Q5TFM2_HUMAN]	1	Not in Control IP
ILF3_612155_Band B	Fibrinogen gamma chain (Fragment) OS=Homo sapiens GN=FGG PE=1 SV=1 - [C9JPQ9_HUMAN]	1	Not in Control IP
ILF3_612155_Band B	Inter-alpha-trypsin inhibitor heavy chain H1 OS=Homo sapiens GN=ITIH1 PE=1 SV=1 - [F8WAS2_HUMAN]	1	Not in Control IP
ILF3_612155_Band B	Inter-alpha-trypsin inhibitor heavy chain H4 (Fragment) OS=Homo sapiens GN=ITIH4 PE=1 SV=1 - [H7C0L5_HUMAN]	1	Not in Control IP
ILF3_612155_Band B	Vimentin (Fragment) OS=Homo sapiens GN=VIM PE=1 SV=1 - [Q5JVS8_HUMAN]	1	Not in Control IP
ILF3_612155_Band B	Splicing factor, proline- and glutamine-rich OS=Homo sapiens GN=SFPQ PE=1 SV=2 - [SFPQ_HUMAN]	3	0.50
ILF3_612155_Band B	Heat shock protein HSP 90-beta OS=Homo sapiens GN=HSP90AB1 PE=1 SV=4 - [HS90B_HUMAN]	4	0.33
ILF3_612155_Band B	Isoform Short of Heterogeneous nuclear ribonucleoprotein U OS=Homo sapiens GN=HNRNPU - [HNRPU_HUMAN]	21	0.05
