# Supplementary figures and images for: NF90/ILF3 is a transcription factor that promotes proliferation over differentiation by hierarchical regulation in K562 erythroleukemia cells
Source: PLoS One. 2018 Mar 28;13(3):e0193126. doi: 10.1371/journal.pone.0193126 (PMC5873942; doi:10.1371/journal.pone.0193126)

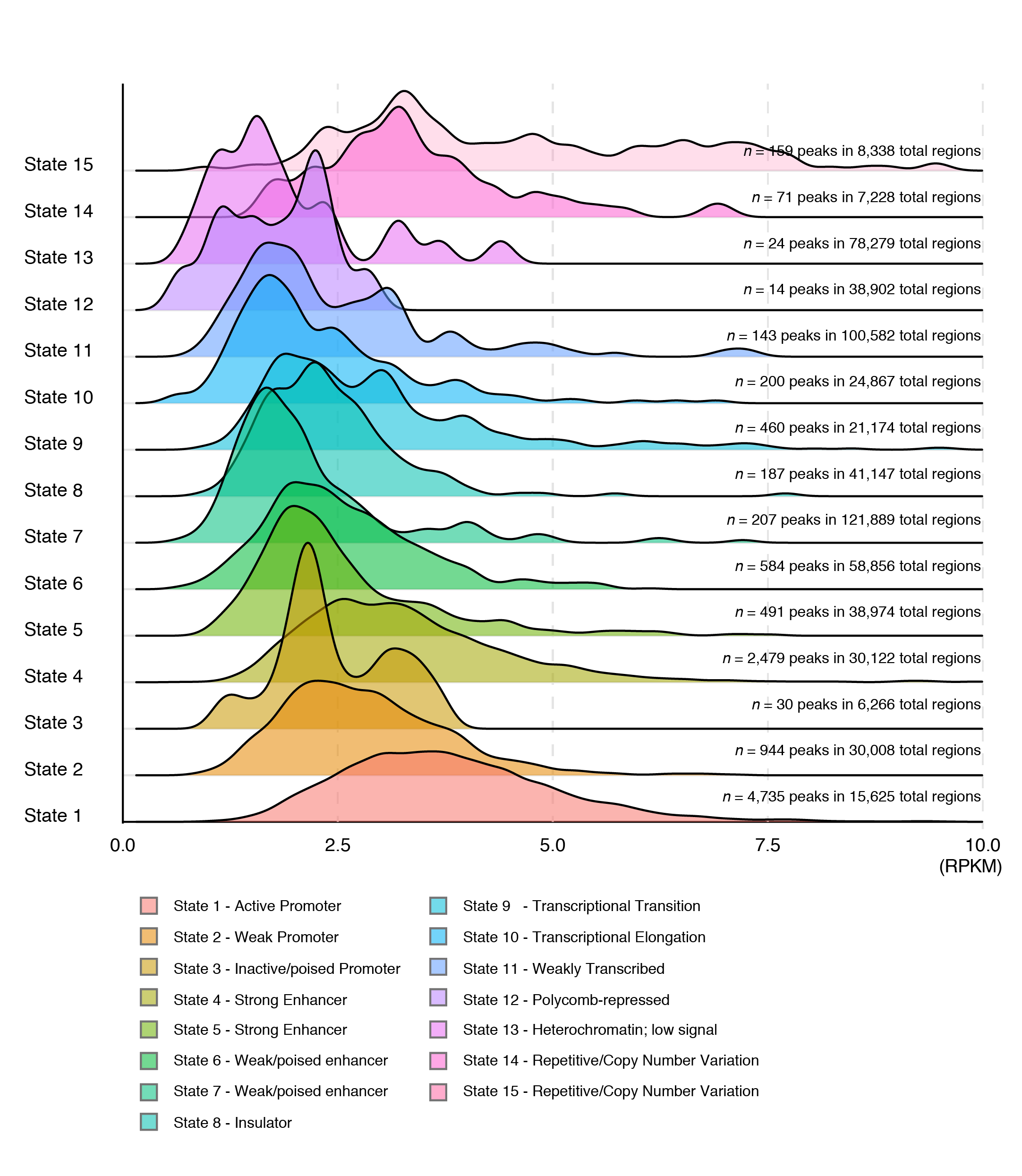

Supplement: S1 Fig — IDR peaks called from NF90/NF110 ChIP-seq experiment in K562 were sorted according to the chromatin state they resided in. The segmented peaks for each of 15 chromatin states were then used to query the ChIP-seq read files to count the number of reads to obtain affinity information for each peak. The resulting distribution of NF90/NF110 occupancy frequencies in different chromatin states were plotted as a histogram. x-axis: Reads Per Kilobase of transcript per Million mapped reads (RPKM). y-axis: 15 chromatin states in which NF90/NF110 peaks resided in. (TIF) [file pone.0193126.s006.tif]

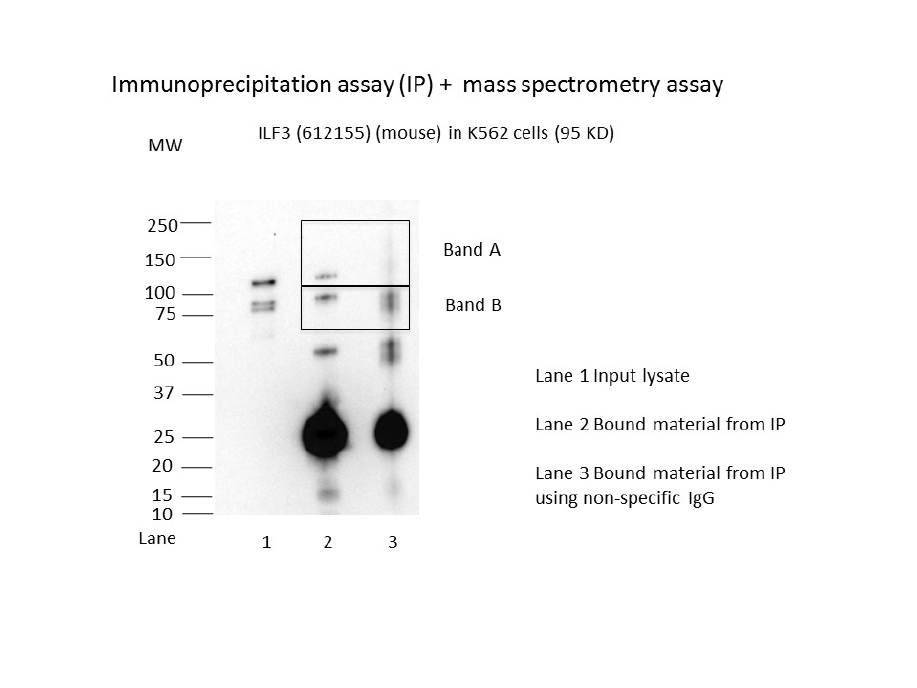

Supplement: S2 Fig — Immunoprecipitation was performed on nuclear extracts from the cell line K562 using antibody against NF90/NF110 (mAb DRBP76; BD 612155). Lane 1: input nuclear lysate. Lane 2: material immunoprecipitated with antibody. Lane 3: material immunoprecipitated using control IgG. Marked bands were excised from gel and subjected to analysis by mass spectrometry. Target molecular weight: 95.338. (TIF) [file pone.0193126.s007.tif]
